# Challenging nurse student selection policy: Using a lifeworld approach to explore the link between care experience and student values

**DOI:** 10.1002/nop2.88

**Published:** 2017-07-16

**Authors:** Janet Scammell, Desiree Tait, Sara White, Michael Tait

**Affiliations:** ^1^ Faculty of Health and Social Sciences Bournemouth University Bournemouth UK; ^2^ Iatrotec Professional Learning Bournemouth UK

**Keywords:** care culture, humanizing care, lifeworld, nurse education, nurse students, selection policy, values

## Abstract

**Aim:**

This study uses a lifeworld perspective to explore beginning students’ values about nursing. Internationally, increasing care demand, a focus on targets and evidence of dehumanized care cultures have resulted in scrutiny of practitioner values. In England, selection policy dictates that prospective nursing students demonstrate person‐centred values and care work experience. However, there is limited recent evidence exploring values at programme commencement or the effect of care experience on values.

**Design:**

Mixed method study.

**Methods:**

A total of 161 undergraduate nursing students were recruited in 2013 from one English university. Thematic content analysis and frequency distribution to reveal descriptive statistics were used.

**Results:**

Statistical analysis indicated that most of the values identified in student responses were not significantly affected by paid care experience. Five themes were identified: How I want care to be; Making a difference; The value of learning; Perceived characteristics of a nurse; and Respecting our humanity. Students readily drew on their *experience of living* to identify person‐centred values about nursing.

**Why is this research needed?**

- There is increasing international concern about uncaring and dehumanized nursing care.
- Undergraduate nurse education plays a part in shaping and reinforcing the values of student nurses.
- Previous studies on the values of student nurses have predominately used a deductive approach and have focused on values development and professional socialization from student to Registered Nurse.
- There is little evidence to support the English policy requirement for care experience before commencing a nursing degree to consolidate caring values.

**What are the key findings?**

- This study updates and supports previous research; beginning students demonstrated a well‐defined understanding of what is required to provide humanized care and how to recognize dehumanized care.
- This study provides insights into the significance of students’ lifeworld prior to exposure to any nurse education in shaping perceptions about nursing and care.
- While perceptions of values were broadly similar across the participants, small differences were noted in relation to age, but not in relation to paid care work experience.

**How should the findings be used to influence policy/practice/research/education?**

- This study calls into question the value of policy imperatives to mandate paid care experience as a criterion of entry into pre‐registration nursing programmes.
- The exploration of lifeworld as an expression of being may provide a deeper understanding of students’ values and be used as a base for further development as students progress through the programme.
- Further research using a lifeworld approach is required to explore how students’ humanized values evolve following exposure to curricular content and practice placement.

## 1 INTRODUCTION

The professional values that inform nursing are recognized internationally through ethical codes that require practitioners to make the care of people their first concern, focusing on respectfulness, compassion, trustworthiness, partnership, competence and safety (ICN 2012; NMC, 2015). Recognizing nursing values that focus on the person and their human rights arguably offers an opportunity to balance the technical and human dimensions of care to achieve high quality (Bentzen, Harsvik, & Brinchmann, 2013; Galvin & Todres, 2013). However recently, the value‐base of nursing has come under scrutiny as criticisms about care quality and dehumanization have emerged internationally (Francis, 2013; McHugh et al., 2013; OECD, 2013). In the United Kingdom (UK), nurse education regulators have responded by developing and refining values‐based recruitment processes; in England, candidates are required to have care experience prior to commencing their course (HEE, 2014a; HEE, 2014b), in line with a recommendation from the Francis Inquiry (2013); other UK countries have taken a less prescriptive approach although work and volunteer experience in general is considered in the selection process in Scotland (NHS Education for Scotland, 2017). The premise for care experience appears to be that selecting recruits whose personal values align with the National Health Service (NHS) (NHS, 2012) will: “ensure that the NHS has the right workforce” (NHS Employers, 2015). Furthermore, gaining “hands on experience” in a nursing assistant role is advocated as a pre‐condition of entry (Francis, 2013: 1539), to expose the potential recruit to role modelling of person‐centred values. However, research evidence concerning nurse student supervision suggests this outcome is no means guaranteed (Chesser‐Smyth, 2006; Jonsen, Melender, & Hilli, 2013). Further this perspective somewhat undermines the innate personal values and life experience that new recruits bring.

This paper focuses on developing a deep understanding of the personal values of students who have been recruited using a values‐based selection strategy as they begin their nursing programme. Values have been described as the ideals and beliefs that individuals hold and express consciously and unconsciously through their actions; values therefore can be used both personally and collectively to set standards of behaviour and practice (Jiménez‐López, Roales‐Nieto, Seco, & Preciado, 2016; Kaya, Işik, Şenyuva, & Kaya, 2016; Rassin, 2008; Rokeach, 1973). Studies of nurse student values are not new (Day, Field, Campbell, & Reutter, 2005; Iacobucci, Daly, Lindell, & Griffin, 2013; Jiménez‐López et al., 2016; Kaya et al., 2016; Mackintosh, 2006; Murphy, Jones, Edwards, James, & Mayer, 2009; Smith, 1992; Watson, Deary, & Lea, 1999), although less research on new entrants prior to any curriculum input is evident. Little is known about how ethical principles (based on values) become part of our “way of being”, nor how they influence day‐to‐day methods of connecting with others in humanized ways. This is significant as literature highlighting poor care quality acknowledged that while individual practitioners professed to holding person‐centred values, the organizational culture had permitted dehumanized care practices (Francis, 2013; Health Service Ombudsman, 2011; Tolson et al., 2011). Clearly knowing values‐based ethical principles is vital but this differs from living them. We contend that values do not exist independently of the person but form part of our way of being. This study is informed by an ontological perspective of embodied lifeworld (Dahlberg & Drew, 1997; Dahlberg, Todres, & Galvin, 2009); it seeks to understand students’ perceptions of their personal values and how they see this in relation to the purpose of nursing, before their exposure to curriculum content and placement experience.

### 1.1 Background

There has been considerable philosophical discourse on the ontological and epistemological traditions and practice of nursing, but central to this debate has been a requirement to place the person at the centre of any discourse (Adams, 2016; Bishop & Scudder, 1990; Edwards, 1998; Zanotti & Chiffi, 2016). In this study an existential view of being human was informed by Heidegger (1962) and served as the starting point for a theoretical framework where nursing can be considered as holistic, complex, intersubjective and context orientated. This challenges the notion of a reductionist and positivistic perspective of being human, where mind and body are viewed as separate and not reflective of the holistic intent of nursing (Benner, 2000; Benner & Wrubel, 1989; Dahlberg & Drew, 1997; Edwards, 1998; Merleau‐Ponty, 1962). This view of being human is reflected in the Nordic tradition of caring sciences (Arnan, Ranheim, Rydenlund, Rytterstrom, & Rehnsfeldt, 2015). In particular, Dahlberg et al. (2009: 266) advocate this perspective for nursing as “we do not live our lives in unrelated compartments” but that mind and body is experienced in a seamless way in everyday life.

Drawing on Husserl's lifeworld theory, Dahlberg et al. (2009) developed a lifeworld perspective of caring science that focuses on the world as it is experienced. This stands in contrast to a biomedical perspective, whereby health care is perceived as part of an objective world that can minimize or even ignore the context where everyday living happens. Reflecting on the target‐driven nature of health care, Galvin and Todres (2013) add that missing from contemporary health care, is sufficient attention to what it means to be human. Todres, Galvin, and Holloway (2009) described a theory of lifeworld‐led care which acknowledges the primacy of people's everyday lives as a context for humanizing practices. This is reflected in their humanizing values framework (HVF), which outlines eight philosophically informed core dimensions of lifeworld‐led care. The HVF can be used by practitioners to reconnect with humanizing and dehumanizing elements in caring systems and interactions (Borbasi et al., 2012).

Central to lifeworld theory is the ontological concept of embodiment, which Draper (2014: 2236) defines as the “experience of living in and through our bodies”. The significance of our bodies in all aspects of everyday life is underestimated beyond their physical properties (body as object); Draper (2014) argued that an embodied understanding (as a way of being) is crucial to our understanding of who we are and therefore the construction of our identity. According to Benner (2000:6), rational and scientific knowledge “is silent about the human experience of illness, recovery and health” although like Carper (1978) she acknowledges that practitioners require both scientific and embodied knowledge to care effectively.

There is some evidence that new recruits commence pre‐registration nurse education with strong caring values (Day et al., 2005; Murphy et al., 2009) but become cynical about their importance when faced with the pressures of clinical practice (Mackintosh, 2006; Murphy et al., 2009). Mackintosh (2006) conducted a descriptive longitudinal study in England of the impact of socialization on students’ views about care and their personal ability to cope with being a nurse. Sixteen participants were interviewed 6 months after programme commencement and again prior to completion. Over time she found a loss in idealism about nursing care due to a process of professional socialization which involved “fitting in” to cope with the realities of the nursing role. She reported that this militated against individuals’ abilities to care. Students’ perceptions at programme commencement were not explored.

Murphy et al. (2009) undertook a longitudinal single cross‐sectional survey study in Wales. Two student cohorts (1^st^ year, *n *=* *80 and 3^rd^ year, *n *=* *94) were involved; first year data were gathered at programme commencement. Using the Caring Behaviours Inventory (Wolf et al., 1994), significantly lower scores were found in third years compared with first years. Contrary to the argument expounded by Francis (2013), higher first year scores (more caring behaviours) were evident among younger students with no prior care experience. Caution is required in interpreting these findings however as two separate cohorts were compared and individuals were not matched; differences may have been related to the cohort. The researchers concluded these results reflected a move from idealized ideas of caring to “a more tempered professional realism” (Murphy et al., 2009: 262). It was proposed that the higher scores in younger students with no care experience might indicate they had internalized lay beliefs from the wider culture about how nurses should behave. One limitation is that the concept of caring was predefined in the survey, whereas the way we live out our values in everyday life depends on specific situations and interconnections.

Another longitudinal study of students entering a 4‐year nursing programme by Kaya et al. (2016) in Turkey used a questionnaire that incorporated the value preference scale, the professional values precedence scale and the nursing professional values scale. The study examined the relationship between a range of predetermined value statements related to social, economic, political, aesthetic, moral, scientific and religious values. While such research is very useful, the focus has been on determining the extent to which ethical principles guide practice and how they may change over time rather than students’ lived experience of values.

In summary, given the use of predetermined inventories it is argued that most of these studies, with the exception of Mackintosh (2006), are reductionist. Studies that explore personal ontology as a way of understanding values as a way of being are less evident. We contend that values are a feature of embodied knowledge. Using a lifeworld perspective, the intent of this study is to explore students’ perceptions of their values about nursing and its purpose when beginning a nursing degree. This approach will yield insights into what is important to participants about nursing and care, primarily from the perspective of human beings, rather than nursing recruits and therefore what is likely to influence their behaviour. By drawing on their lived experience as consumers of health care and for some, experiences of paid care work, it is anticipated that an understanding of values as a way of being, will inform pedagogical strategies to support and maintain their expression in everyday nursing practice.

## 2 THE STUDY

### 2.1 Aim

Using a lifeworld perspective, this study aimed to explore students’ perceptions of their values about nursing and its purpose when beginning a nursing degree. The research questions asked:

- How do new nursing students describe their values when supported to use a lifeworld perspective?
- Do age and previous care experience effect novice students’ perception of their values?

### 2.2 Design

A mixed method design was used. The questionnaire used a Values Clarification Exercise (VCE), a tool designed to access and clarify the values and beliefs that we hold about something (Manley, 2000). It commences with a main stem that describes the purpose of something and then moves onto how this is achieved and the associated barriers and enablers. Drawing on Manley's (2000) VCE process, students were asked to respond to six value statements:

- I believe the purpose of nursing is to…
- I believe this purpose can be achieved by…
- The factors that inhibit or enable this to be achieved include…
- I want to be a nurse because…
- I feel valued as a person when…
- I do not feel valued as a person when…

Participants were invited to provide a short free‐text response to each VCE statement (Appendix 1). The purpose of using this approach was to obtain qualitative data based on participants’ personal perspective rather than a prescribed list of value statements. Space was limited to enable ease of completion and to place some limit on the extent of data generated for logistical and pragmatic reasons.

### 2.3 Sample

One cohort adult‐field nursing students (*n *=* *180) were invited to complete the VCE. About 89% (*n *=* *161) agreed to take part.

### 2.4 Data collection

Students were invited by a member of the research team [SW] to complete a paper‐based anonymized questionnaire (the VCE) on day 1 of their programme. These were collected on completion and the students informed that they would be returned to them for use in a teaching session the following week; at the end of that session, the students were asked to either opt to keep their VCE or return it to the unit lecturer for inclusion in the study. Participation in the study was voluntary. The unit lecturers forwarded the returned VCEs to the research team.

### 2.5 Ethical considerations

University research ethics approval was secured and a participant information sheet was sent to each student prior to programme commencement. Informed consent was gained and completed anonymized VCEs were kept in locked storage and accessed only by the research team.

### 2.6 Data analysis

Data were in the form of free‐text written responses to each VCE statement. Data ranged from a few words to two sentences per statement. Qualitative content analysis was used to analyse the VCE responses. This is a way of analysing written, verbal or visual communication messages (Cole, 1988). It is an exploratory and empirically grounded method (Krippendorff, 2013) allowing the researcher to test theoretical issues to enhance the understanding of data. An inductive manifest content analysis (Graneheim & Lundman, 2003) was used which involved categorization of the meaning units from analysis of the six value clarification statements to identify sub‐themes and overarching themes [JS, SW, DT]. Statistical analysis using SPSS was used to identify the frequency of responses in each sub‐theme [MT] and to compare the effect of previous paid care experience and age on participants’ value statements (Pallant, 2013).

### 2.7 Validity and reliability

Internal validity of the data was achieved by using three of the research team [JS, DT, SW] to independently analyse the statements and compare their findings across the sample. Reliability and consistency was established through peer review until there was an agreed understanding of the meaning units, sub‐themes and themes. A unique code was assigned to each completed VCE, which protected identity but gave the researchers opportunity to trace meanings of statements across categories and themes (Morse, 2015). This allowed the researchers to code and map an individual audit trail for each participant [MT]. Table 1 summarizes the demographic data and codes used for each variable. External validity was demonstrated by the large sample size and the high response rate of the participants.

**Table 1:** Demographic data for respondents to the values clarification questionnaire

Variables	Response	Code	Frequency	Per cent
Prior experience of paid caring	Yes	Y	86	53.4
	No	N	46	28.6
	No response	NA	29	18
Age range (yrs)	<20	1	46	28.6
	20–29	2	77	47.8
	30–39	3	21	13
	40+	4	15	9.3
	No response	–	2	1.2
Gender	Female	F	147	91.3
	Male	M	12	7.5
	No response	–	2	1.2

## 3 FINDINGS

Of the 89% (*n *=* *161) of students who agreed to take part, 53.4% (*n *=* *86) had prior paid caring work experience and 28.6% (*n *=* *46) did not, while 18% (*n *=* *29) did not reply to this question. About 91.3% (*n *=* *147) were females and 7.5% (*n *=* *12) males, while 1.2% (*n *=* *2) left this question blank. The study participants varied in age from 18–52 years with the majority aged between 18–29.

### 3.1 Initial content and statistical analysis of student responses to the values clarification questionnaire

From initial content analysis of the responses to the six value statements, several sub‐themes were identified. The number and percentage of respondents who included each theme in their responses was then calculated. Finally, these themes were ranked in order of frequency for each value statement (Table 2).

**Table 2:** Sub‐themes identified by content analysis of student responses to statements in the values clarification questionnaire and numbers of students whose responses included each sub‐theme

Value clarification statement	Sub‐themes	Number	Per cent
The purpose of nursing is to…	Be caring and compassionate	86	53.4
	Promote health and well‐being	79	49.1
	Provide holistic care	51	31.7
	Promote comfort and minimize suffering	29	18
	Care for vulnerable people	25	15.5
	Provide high quality care	19	11.8
	Make a difference	12	7.5
	Communicate well with people	10	6.2
	Make clinical judgements	4	2.5
	Provide a safe environment	3	1.9
This purpose can be achieved by…	Being caring and compassionate	67	41.6
	Having the right knowledge and skills and CPD	61	37.9
	Giving holistic care	45	28
	Good communication	39	24.2
	Having courage and commitment	24	14.9
	Being professional	21	13
	Team working	16	9.9
	Showing dignity and respect	9	5.6
The factors that inhibit or enable the achievement of this purpose include…	Resources	54	33.5
	Caring and compassion	53	32.9
	Learning and knowledge	53	32.9
	Communication	50	31.1
	Team working	29	18
	Holistic care	18	11.2
	Being professional	10	6.2
	Media	7	4.3
I want to be a nurse because…	I want to make a difference	85	52.8
	I am caring/compassionate	58	36
	I want to learn	34	21.1
	I believe I have the passion and qualities it takes	19	11.8
	Of the career opportunities	17	10.6
	It's challenging work	15	9.3
I feel valued as a person when…	I'm listened to and respected	83	51.6
	I'm being acknowledged	64	39.8
	I'm making a difference	46	28.6
I do not feel valued as a person when…	I'm being ignored	87	54
	I'm not respected or listened to	50	31.1
	I'm being undermined and lied to	21	13
	I'm being pre‐judged	16	9.9
	I feel helpless	14	8.7
	I don't feel part of the team	4	2.5

In response to the value statement: “The purpose of nursing is to…”, the sub‐theme most frequently identified was “be caring and compassionate” (*n *=* *86). This represents 53.4% of the total number of respondents. Other frequently identified sub‐themes included “promoting health and well‐being” (49%), “providing holistic care” (31.7%) and “promoting comfort and minimising suffering” (18%). Only three students (1.9%) included the sub‐theme “provide a safe environment” in their response. When asked to respond to the value statement “I want to be a nurse because…”, the most frequently identified sub‐themes were: “I want to make a difference” (52.8%) and “I am caring/compassionate” (36%). In response to statements exploring when the students “felt valued” or “not valued”, the most frequent sub‐themes identified were 51.6% felt valued when they were “listened to and respected” and 54% did not feel valued when they were “being ignored”. Some sub‐themes were identified in the responses to more than one value statement. For example, the theme “caring and compassionate” was included by students in their responses to four of the six value statements: The purpose of nursing is…; This purpose can be achieved by…; The factors that inhibit or enable the achievement of this purpose include…; and I want to be a nurse because…

### 3.2 The effect of experience in health care on student responses to the values clarification questionnaire

Eighty‐six respondents indicated that they had paid care experience and 46 said that they had none. Twenty‐nine other students did not respond to this question. To determine whether experience affected the students’ responses to the questionnaire, the data for those that had given their level of experience (*n *=* *132) were further analysed. For each sub‐theme shown in Table 2, the percentages of students who had care experience or had no care experience and who included that sub‐theme in their response were calculated (Table 3). For example, 52.3% of students who had care experience included the sub‐theme “caring and compassionate” in their responses to the value statement The purpose of nursing is… and 54.3% of students with no care experience also included that sub‐theme in their responses.

**Table 3:** Effect of experience in health care on sub‐themes detected in student responses to the values questionnaire

Value statement	Sub‐themes identified	Experience %	No experience %	Chi‐square χ^2^(1)	Significance *p*	Phi coefficient
The purpose of nursing is to…	Be caring and compassionate	52.3	54.3	0.002	.969	
	Promote health and well‐being	47.7	43.5	0.077	.781	
	Provide holistic care	26.7	34.8	0.584	.445	
	Promote comfort and minimize suffering	16.3	26.1	1.255	.263	
	Care for vulnerable people	18.6	13.0	0.327	.567	
	Provide high quality care	10.5	13.0	0.025	.875	
This purpose can be achieved by…	Being caring and compassionate	38.4	50.0	1.217	.27	
	Having the right knowledge and skills and CPD	39.5	28.3	1.206	.272	
	Giving holistic care	26.7	32.6	0.257	.612	
	Good communication	23.3	30.4	0.476	.49	
	Having courage and commitment	9.3	23.9	4.074	.044	−0.198
	Being professional	11.6	21.7	1.662	.197	
The factors that inhibit or enable the achievement of this purpose include…	Resources	37.2	15.2	5.947	.015	0.23
	Caring and compassion	29.1	41.3	1.506	.22	
	Learning and knowledge	32.6	37.0	0.099	.753	
	Communication	27.9	34.8	0.385	.535	
	Team working	22.1	19.6	0.013	.908	
	Holistic care	10.5	15.2	0.268	.605	
I want to be a nurse because…	I want to make a difference	51.2	58.7	0.415	.52	
	I am caring/compassionate	38.4	32.6	0.217	.641	
	I want to learn	24.4	15.2	1.018	.313	
	I believe I have the passion and qualities it takes	14.0	13.0	0	1	
	Of the career opportunities	9.3	17.4	1.16	.281	
I feel valued as a person when…	I'm listened to and respected	46.5	58.7	1.326	.25	
	I'm being acknowledged	43.0	39.1	0.061	.805	
	I'm making a difference	34.9	17.4	3.661	.056	
I do not feel valued as a person when…	I'm being ignored	58.1	41.3	2.763	.096	
	I'm not respected or listened to	27.9	41.3	1.877	.171	
	I'm being undermined and lied to	5.8	23.9	7.596	.006	−0.264
	I'm being pre‐judged	10.5	13.0	0.025	.875	

The statistical significance of the difference between the sub‐themes identified in the responses given by students with and without experience in health care was analysed in SPSS. For some sub‐themes, the chi‐square value obtained was not valid because the number of students including that sub‐theme in their response was too low. These sub‐themes have therefore been excluded from Table 3. For those sub‐themes where a valid chi‐square value could be calculated, most had a significance value (*p*) above .05 (Table 3). This indicates that the students’ experience in health care had no significant effect on the inclusion of those sub‐themes in their responses to the value statements. For example, if the number of students whose response to the value statement “The purpose of nursing is to… included be caring and compassionate”, there was no significant difference between students who had care experience (52.3%) and those who had no experience (54.3%).

Only three sub‐themes had a significance value of 0.05 or less: (1) “This purpose can be achieved by… having courage and commitment” (*p *=* *.044); (2) “The factors that inhibit or enable the achievement of this purpose include… resources” (*p *=* *.015); and (3) “I do not feel valued as a person when… I'm being undermined and lied to” (*p *=* *.006). This indicates that there was a relationship between care experience and the inclusion of those sub‐themes in the students’ responses. When the phi coefficients for these sub‐themes were calculated (Table 3), two were very low (−0.198 and −0.264), indicating that the effect for these themes was very small. The effect was large for the theme “The factors that inhibit or enable the achievement of this purpose include… resources” (phi = 0.23).

### 3.3 The effect of age on student responses to the values clarification questionnaire

Of the 161 students who completed the questionnaire, 46 gave their age as less than 20, 77 were 20–29, 21 were 30–39 and 15 were 40 or more (two gave no response). To facilitate analysis, the results from the final two age groups were combined to give a group of 36 students who were aged 30 or more (Table 4).

**Table 4:** Effect of age on themes identified in student responses to the values questionnaire

Value statement	Sub‐themes identified	Age <20%	Age 20–29%	Age 30 + %	Pearson chi‐square χ^2^(2)	Significance *p*	Cramer's V
The purpose of nursing is to…	Be caring and compassion	60.9	50.6	50.0	1.433	.489	
	Promote health and well‐being	43.5	50.6	52.8	0.85	.654	
	Provide holistic care	50.0	31.2	11.1	14.075	.001	0.298
	Promote comfort and minimize suffering	19.6	16.9	19.4	0.184	.912	
	Care for vulnerable people	8.7	20.8	13.9	3.291	.193	
This purpose can be achieved by…	Being caring and compassionate	43.5	45.5	33.3	1.526	.466	
	Having the right knowledge and skills and CPD	39.1	39.0	30.6	0.856	.652	
	Giving holistic care	32.6	26.0	27.8	0.631	.729	
	Good communication	26.1	19.5	33.3	2.628	.269	
	Having courage and commitment	17.4	15.6	11.1	0.649	.723	
The factors that inhibit or enable the achievement of this purpose include…	Resources	26.1	36.4	38.9	1.86	.395	
	Caring and compassion	30.4	36.4	30.6	0.617	.735	
	Learning and knowledge	32.6	35.1	27.8	0.592	.744	
	Communication	37.0	27.3	33.3	1.33	.514	
	Team working	10.9	23.4	16.7	3.098	.212	
I want to be a nurse because…	I want to make a difference	43.5	59.7	50.0	3.206	.201	
	I am caring/compassionate	45.7	35.1	27.8	2.913	.233	
	I want to learn	15.2	23.4	19.4	1.206	.547	
I feel valued as a person when…	I'm listened to and respected	52.2	51.9	50.0	0.047	.977	
	I'm being acknowledged	37.0	41.6	41.7	0.292	.864	
	I'm making a difference	28.3	31.2	22.2	0.968	.616	
I do not feel valued as a person when…	I'm being ignored	39.1	62.3	55.6	6.286	.043	0.199
	I'm not respected or listened to	34.8	33.8	19.4	2.837	.242	

To determine whether age affected the students’ responses to the questionnaire, the data for those who had given their age (*n *=* *159) were further analysed. For each sub‐theme shown in Table 2, the percentages of students in each age range who included that sub‐theme in their response were calculated (Table 4). For example, 60.9% of students who were aged less than 20 included the theme “be caring and compassionate” in their response to the value statement: “The purpose of nursing is to…”, whereas 50.6% of students aged 20–29 and 50% of students aged 30 or more included that sub‐theme in their response.

The statistical significance of the difference between the sub‐themes identified in the responses given by students in the three age bands was analysed in SPSS using chi‐square tests. For some sub‐themes, the chi‐square value obtained was not valid because the number of students including that sub‐theme in their response was too low. These sub‐themes were excluded from Table 4.

For those sub‐themes where a valid chi‐square value could be calculated, most had a significance value (*p*) above .05 (Table 4), indicating that there was no significant relationship between the students’ age and the inclusion of those sub‐themes. Only two sub‐themes had a significance value of 0.05 or less: (1) “The purpose of nursing is to… provide holistic care” where older students were less likely to include this theme in their responses; and (2) “I do not feel valued as a person when… I'm being ignored” where students aged 20–29 were most likely to include this theme in their responses. The Cramer's V value for the first of these themes was 0.298, indicating that the effect was medium to large. For the second of the themes, it was 0.199, indicating that the effect was small to medium in size.

### 3.4 Overarching themes emerging from content analysis

To allow further analysis of the student free‐text responses, the sub‐themes shown in Table 2 were collapsed into five overarching themes:

- respecting our humanity;
- how I want care to be;
- perceived characteristics of a nurse;
- making a difference; and
- the value of learning.

The theme “respecting our humanity” demonstrates a valuing of mutual regard for persons as individuals—students, colleagues and care‐recipients:

When I am respected for what I can contribute and am given the opportunity to learn and grow as a person [119F2NA]

(See Table 1 for explanation of participant identifiers).

someone says thank you or gives me an opportunity to show my ability as part of a team [71F2Y]

On the other hand, comments acknowledge the negative impact when this is not evident:

When I'm spoken down to, belittled, feel degraded [160F2N]

The second overarching theme “how I want care to be”*,* captures participants ideal of what nursing should be:

being compassionate, caring and empathic to every individual's need [5F1N]

to be there for the patient when they are at their most vulnerable to protect, care and try to get them independent again [154M2Y]

Although new recruits, they could recognize factors that inhibited or enhanced achievement of this ideal, particularly in the context of caring and compassion, holistic care, communication and resources:

poor communication, lack of understanding, compassion and empathy can inhibit nursing care [132F2NA]

In the third theme “perceived characteristics of a nurse”, participants shared their passion and belief about the personal qualities of a nurse. Historic and personal influences (DH (Department of Health), 2012) were evident:

work hard, be determined, dedicated and courageous [81M1N]

I am very passionate about being a nurse and due to lots of personal experience as a patient I feel I can put myself in their shoes [82F1N]

“Making a difference” was the fourth theme and represented a personal motivation to have a positive impact on the service users in their care:

I want to deliver the highest standards of care [141NA]

I am able to bring a smile to someone's face, it doesn't have to be a big thing [75F2Y]

The final theme that emerged was the “value of learning” and reflected ideas about importance of learning and its impact on care quality as well as personal development:

continually enhancing knowledge and understanding to ensure the best possible care [65F2N]

In summary, the overarching themes capture the innate values and beliefs of new nursing recruits concerning nursing. This is reflected across the themes but, in particular: “how I want care to be”; “making a difference”; “the value of learning”; and “perceived characteristics of a nurse”. The theme: respecting our humanity illustrates how the recruits appear to draw on their life experiences to present what it means to be valued and devalued, highlighting a fundamental aspect of what it means to be human. This is also evident in the themes: “how I want care to be”; “making a difference”; and “the perceived characteristics of a nurse”.

## 4 DISCUSSION

The findings update and support previous research that nurse students hold person‐centred values on entry to nurse education (Murphy et al., 2009). For example, the phrase most frequently used to describe the purpose of nursing was “to be caring and compassionate”, related to being holistic, promoting comfort and alleviating suffering. However, the lifeworld approach seemed to elicit meanings about being a nurse and the impact of nursing, ideas about their approach to care, rather than tasks, what Rolfe (2009) termed “being caring” as opposed to “doing caring”.

Concerns about care quality (Francis, 2013; McHugh et al., 2013; OECD, 2013) were less prevalent at the time of previous studies and concurrent media focus on compassionate practice may have had an impact, particularly on these students. However by adopting an embodied approach, the focus was on nursing as a way of being through exploring meaning in everyday lived experience, the students revealed personal knowledge (Carper, 1978) derived from their personal belief systems. For example, the participants were not only asked to think about how they valued others but also about experiences of how *they* felt valued or not; this revealed that “being or not being listened to and respected” was the most frequently cited factor. In contrast to other studies, the lifeworld approach taps into what Draper (2014) terms a more subjective view of nursing and care through consideration of everyday lived experience; these findings indicate that making a meaningful and authentic connection with others is as important for care staff as service users to enable humanized care.

Contrary to Murphy et al. (2009) findings that student recruits with no care experience scored more highly in caring behaviours than older recruits with care experience, our study found that new students with care experience did not express person‐centred values any differently than those who did not. The impact of care experience had no significant impact in this study: 52.3% (students with care experience) versus 54.3% (students with no care experience) included the sub‐theme “caring and compassionate” in their responses to “The purpose of nursing”. This could be accounted for by changing attitudes in practice and society; the fundamental importance of person‐centred care is more prominent in health care today in the aftermath of the Francis (2013). Furthermore, the concept of caring was predefined in the Murphy et al. (2009) study, whereas a lifeworld approach recognizes that the way we live out our values depends on specific situations. It is interesting therefore that the only significant difference to emerge between the two groups was the perception of the care experience group that a lack of resources can inhibit achieving the purpose of nursing.

These findings challenge the policy imperative in England that all nurse recruits should have care experience prior to entering nurse education, specifically work as a care assistant (Francis, 2013; HEE, 2014b). Evidence in support of its link to values is lacking and interestingly this mandate is not reflected internationally. Lovegrove and Griffin (2014) in their evaluation of pre‐nursing degree care experience pilot found that participants increased in confidence but no evidence of increased caring values emerged. Similarly, Snowden et al. (2015) found that previous care experience was not associated with higher emotional intelligence, a factor frequently linked with caring values. Our study adds that regardless of care experience, students at the beginning of their programme had a well‐defined understanding of what is required to provide humanized care and how to recognize dehumanized care. Rather than requiring pre‐nurse education care experience, it may be more important to help students identify and “own” humanizing values at course commencement and then focus on supporting resilience to maintain these when exposed to nursing practice (Curtis, Horton, & Smith, 2012).

Turning to age, Murphy et al. (2009) also found significantly higher first‐year caring behaviour scores in those under 26 also with no care experience and concluded this reflected idealized ideas nursing. In contrast, statistical analysis of the data obtained in our study indicated that there was no significant relationship between students’ age and the inclusion of most sub‐themes identified in the students’ responses to the values clarification questionnaire. However, for two sub‐themes (“purpose of nursing” and “not feeling valued”), there was an effect but this was small to medium in size. Older students highlighted the importance of holistic care more than younger students perhaps reflecting a greater diversity of life experience.

Our study adds to the body of knowledge concerning the values of nurse recruits, providing insights into the significance of students’ lifeworld prior to exposure to any nurse education and professional socialization. The focus on the ontology of nursing through the values clarification questionnaire elicited data around what it means to be a nurse from the perspective of new recruits. Dahlberg and Drew (1997:312) wrote that the aim of lifeworld research is “to discover and articulate the meaning that is implicit in experience and then to base actions on that understanding”. This approach encouraged participants to draw on their lifeworld to respond to the questions, as health service users and in some cases care givers but most significantly as human beings.

In summary, the overarching themes emerging from the study represent the innate values of nurse recruits concerning what it is to be human as revealed through their perceptions of nursing. Galvin and Todres (2013) highlighted that attending to humanizing values given the increasingly technical and specialized nature of care is essential to counter dehumanized care practices. Through the lens of lifeworld, it has been possible to highlight nurse recruits embodied perceptions of nursing, largely untainted by professional socialization. The values to emerge reveal a holistic perspective on being human. The centrality of meaningful relationships is key, with service users and colleagues among others, indicating the importance of intersubjectivity that is “how we are in a world with others” (Galvin & Todres, 2013: 28). This appeared to be central to the expression of humanizing values; the participants wanted to empathize with clients and connect with them to make a difference for the better. Equally they needed to feel valued for what they had to offer and keenly expressed a sense of being devalued when ignored or even lied to. These examples of intersubjectivity illustrated humanizing and dehumanizing behaviour. These new recruits “knew” this regardless of prior exposure to nursing. Clearly any curriculum would need to nurture these insights to prevent the incipient cynicism described by Mackintosh (2006) taking root and effecting care delivery.

### 4.1 Limitations

Students were invited to complete the questionnaire through an online request before meeting lecturing staff, reducing the risk of the power differential having an impact on the students’ responses. However, perceived pressure to respond positively and complete the questionnaire would have been high, as they had just started the programme. It can be argued however that social desirability bias was reduced by the use of indirect questioning and the use of open‐ended questionnaire statements (Fisher, 1993). External validity was demonstrated by the large sample size (*n* = 161) and the high response rate of the participants (89%). Although findings may be generalized to similar populations of beginning undergraduate nursing students, caution is required as participants were drawn from one university and one cohort.

## 5 CONCLUSION

Research about the values of nurse recruits in the last decade mainly concerns empirical and ethical ways of knowing. In contrast, this study focuses on personal ontology as a way of understanding values. The study captured something of the human experience of entering nursing as a non‐professional, from new recruits not yet immersed in professional language and values. It could be argued that these findings add rich qualitative data to compliment that yielded from studies where value statements are predefined in surveys and then measured (e.g. Kaya et al., 2016). Furthermore, the embodied approach used in this study revealed some new insights about the effect of care experience and age and calls into question the value of policy imperatives to mandate paid care experience as a criterion of entry into pre‐registration nursing programmes. By using a lifeworld perspective, this study has provided a unique insight into students’ values on entry to the nursing programme. Further longitudinal research throughout the nurse education programme and beyond is recommended to explore whether students’ humanized values are maintained or change following exposure to curricular content and practice placement experience.

## CONFLICT OF INTEREST

No conflict of interest has been declared by the author(s).

## AUTHOR CONTRIBUTIONS

All authors have agreed on the final version of the paper and meet at least one of the following criteria (based on those recommended by the ICMJE):

- substantial contributions to conception and design, acquisition of data, or analysis and interpretation of data
- drafting the article or revising it critically for important intellectual content.

## APPENDIX 1

### Values Clarification Questionnaire

**Table:**

Tutor Group:	Gender:	Age:
**Paid care work experience—yes/no** (please circle the answer that applies).		
**Date:**		

A Values Clarification Exercise is a simple exercise designed to help us clarify the values and beliefs we hold about something. Nursing is one of a number of caring sciences and therefore it is important that we explore our personal values and beliefs because these influence our behaviour. Through making explicit our values and beliefs, we are taking the first steps to making them a reality in the way we work. Manley (2000) argued that “a match between what we say we believe and what we do is one of the hallmarks of effective individuals, teams and organisations.”

**Table:** Please complete the statements below:

Statement	
I believe the purpose of nursing is …	
I believe this purpose can be achieved by …	
I believe the factors that may inhibit or enable this purpose to be achieved include …	
I want to be a nurse because …	
I feel valued as a person when …	
I do not feel valued as a person when …	

**Thank you. Please note that it is intended that data from this questionnaire will be used as part of a study investigating nurse student values (See Participant Information Sheet). Taking part is voluntary; if you do NOT want your VCE included in the study, please do not return this to your unit teacher at the end of the seminar session.**

**Reference:** Manley, K. (2000) Organisational culture and consultant nurse outcomes: part 1 organisational culture. *Journal of Nursing in Critical Care*, 5 (4): 179.
